# Medicaid Home and Community-Based Services Initiation and Acute Services Use

**DOI:** 10.1001/jamahealthforum.2026.0206

**Published:** 2026-03-27

**Authors:** Emmaline Keesee, Chanee D. Fabius, Jennifer Kim, David Stevenson, Laura M. Keohane

**Affiliations:** 1 Department of Health Policy, Vanderbilt University, Nashville, Tennessee; 2 Department of Health Policy and Management, Johns Hopkins University Bloomberg School of Public Health, Baltimore, Maryland; 3 Vanderbilt University School of Nursing, Nashville, Tennessee

## Abstract

**Importance:**

For more than a decade, Medicaid has funded the majority of long-term services and supports through home and community-based services (HCBS). Whether access to Medicaid HCBS may affect dual-eligible beneficiaries’ use of Medicare-covered medical services is not well understood.

**Objective:**

To determine whether Medicaid HCBS initiation is associated with changes in acute services use and medication fills.

**Design, Setting, and Participants:**

This cohort study used Southern Community Cohort Study data linked to Medicare and Medicaid claims to identify a cohort of older adults residing across 11 southeastern states, largely recruited from community health centers between 2002 and 2009. The sample included older adults with 12 months of continuous traditional Medicare or Medicare Advantage enrollment centered on first month of Medicaid HCBS initiation. All analyses were conducted from spring 2023 to fall 2025.

**Exposure:**

Initiation of Medicaid HCBS between 2006 and 2018. HCBS was identified by presence of a Medicaid personal care claim or 1915(c) waiver enrollment or claim.

**Main Outcomes and Measures:**

Inpatient discharges, emergency department (ED) use, and unique prescription drugs filled estimated using linear regressions with an event study structure. Event study models with person and year fixed-effects estimated changes in person-month probability of ED use and inpatient discharge and unique drugs filled in the 6 months before and after initiation of HCBS. Subanalyses estimated differences by pre-HCBS Medicaid enrollment status, Medicaid waiver vs state plan HCBS use, Alzheimer disease and related dementia, and diabetes diagnosis.

**Results:**

In a sample of 1218 new HCBS users (75% female, 77% Black; mean [SD] age, 70.5 [7.5] years), Medicaid HCBS initiation was associated with a decrease in probability of ED use (−2.70 percentage points; 95% CI, −4.18 to −1.22 percentage points) and a decrease in probability of inpatient discharge (−2.63 percentage points; 95% CI, −3.75 to −1.51 percentage points). These differences represent a 24% decrease in the within-person probability of any ED use and a 32% decrease in inpatient discharge down from adjusted pre-HCBS probabilities of 11.4% and 8.1%. Event-month trends demonstrated discontinuity at HCBS initiation and reduced the probability of acute services use that was maintained 6 months thereafter. The number of unique drugs filled steadily increased before and after HCBS, with no discontinuity observed at HCBS initiation.

**Conclusions and Relevance:**

In this study among a large cohort of older adults with low income across the southeastern US, Medicaid HCBS was associated with a persistent decrease in acute services use. Future research should explore additional outcomes to better inform policies that can improve HCBS outcomes and an understanding of its tradeoffs with acute services use.

## Key Points

**Question:**

Is initiation of Medicaid home and community-based services (HCBS) associated with changes in acute services use?

**Findings:**

In this cohort study among 1218 older adults across 11 states, Medicaid HCBS initiation was associated with a 24% decrease in within-person probability of emergency department use and a 32% decrease in inpatient discharge.

**Meaning:**

Results of this study suggest that, among older adults with unmet need for Medicaid-funded long-term services and supports, initiation of Medicaid HCBS may reduce reliance on costly acute care services.

## Teaser

This cohort study reports on the association of initiation of Medicaid home and community-based services with use of acute services.

## Introduction

Over the last 4 decades, the US federal government has incentivized rebalancing Medicaid long-term services and supports (LTSS) away from nursing homes, a mandatory Medicaid benefit, to home and community-based services (HCBS), a voluntary benefit.^1^ As of 2022, approximately 2.5 million dually eligible beneficiaries used Medicaid HCBS to assist with personal care and home health needs.^2^ Whether access to Medicaid HCBS may affect dual-eligible beneficiaries’ use of Medicare-covered medical services is not well understood.

Frail older adults living at home report high levels of unmet need for assistance with activities of daily living^3^ and associated greater use of emergency department (ED) and inpatient care.^4,5^ Providing support for daily activities through Medicaid HCBS may reduce the use of acute medical care, but existing evidence is limited to specific states and care contexts.^6,7,8^ HCBS may also prevent unplanned hospitalizations or ED visits for dual-eligible beneficiaries through better chronic disease management, including monitoring symptoms, medication reminders, and help getting to medical appointments. However, little evidence exists on the mechanisms by which HCBS may improve outcomes. HCBS may not be sufficient to address the needs of individuals with complex conditions as it is rarely provided continuously and personal care assistants cannot administer medications. There is thus reason to believe that needs of individuals with complex conditions exceed the capacity of HCBS.

Medicaid programs vary widely in the scope and intensity of HCBS services offered,^9^ potentially influencing whether recipients have improved outcomes with HCBS initiation. Most states offer HCBS through 1915(c) waivers to individuals with income up to 300% of the Supplemental Security Income limit who require a nursing home level of care.^10^ Waivers commonly provide multiple services, including personal care, meal delivery, case management, respite, and adult day care. However, waiver programs are not an entitlement benefit. Even if older adults are already enrolled in Medicaid, they may experience delays in HCBS initiation while documenting eligibility for waiver benefits or navigating waiver waiting lists. HCBS benefits offered under a Medicaid state plan as an entitlement may be more readily available to older adults who already have Medicaid coverage. Thirty-four states offer personal care services as part of their state plan. Policymakers need better evidence on how HCBS benefit design and Medicaid coverage status before receiving HCBS might influence outcomes, especially for those with complex conditions. The wider breadth of services under a waiver may be more effective in improving outcomes than state plan personal care services, but individuals receiving waiver services after a waiting period may experience adverse health outcomes associated with delays in HCBS access.

To better identify how Medicaid HCBS may address the unmet needs of older adults, we analyzed whether initiating HCBS for the first time, either through a 1915(c) waiver or state plan personal care, was associated with changes in acute services use (inpatient discharges and ED visits) among Medicare enrollees. We observed within-person changes over time and their persistence in a large multistate cohort that allowed assessment of heterogeneity across subgroups. To assess whether HCBS affects medication use in ways that might affect acute services use, we examined the number of drugs filled by participants before and after HCBS initiation.

## Methods

### Data and Sample

This cohort study used Southern Community Cohort Study (SCCS)^11^ data linked to Medicare and Medicaid claims. The SCCS enrolled a cohort of adults ages 40 to 79 years with primarily low income between 2002 and 2009. It recruited from community health centers (86%) and via mail-based population sampling (14%) across 12 southeastern states (Arkansas, Alabama, Florida, Georgia, Kentucky, Louisiana, North Carolina, South Carolina, Mississippi, Tennessee, Virginia, West Virginia).^12^ The baseline survey assessed demographic characteristics. Linked Medicare and Medicaid claims documented individuals’ diagnostic information from 2003 to 2018 and Medicaid HCBS use from 2006 to 2018. Data files included the Medicare Master Beneficiary Summary File, MedPAR records on inpatient and skilled nursing facility use, outpatient and carrier claims, Medicare Part D prescription drug event data, Minimum Data Set nursing home assessments, and Medicaid Analytic eXtract (MAX) and Transformed Medicaid Statistical Information System (T-MSIS) Analytic Files (TAF) claims and enrollment data. The SCCS was approved by the Vanderbilt University Institutional Review Board. All analyses were conducted from Spring 2023 to Fall 2025 in SAS version 9.4 (SAS Institute) and Stata version 14 (StataCorp) statistical software. This analysis adhered to the Strengthening the Reporting of Observational Studies in Epidemiology (STROBE) reporting guideline.

Monthly HCBS use was identified between 2006 and 2018 according to 1915(c) enrollment or the presence of at least one 1915(c) claim or personal care claim (details in eMethods in Supplement 1). Our sample included SCCS participants with 6 months of continuous Medicare coverage before and after their first observed month of HCBS use. Thus, all participants initiated Medicaid HCBS between July 2006 and July 2018 after at least 6 months without Medicaid HCBS use. If a participant had multiple HCBS episodes, we only included the first episode. To focus on individuals who consistently used HCBS, we excluded individuals with more than a 1-month gap in HCBS use in the 6 months post–HCBS initiation. We excluded individuals who changed Medicare coverage type (traditional vs Medicare Advantage) during the 6-month pre- or post-HCBS periods. All participants survived to at least age 65 years by 2020, although they may have been younger than 65 when they started HCBS. After exclusions, our sample included participants across 11 states (Tennessee was excluded) (eFigure 1 in Supplement 1).

### Variables

Two measures assessed whether HCBS initiation was associated with acute services use: any ED use (yes or no), including outpatient visits and observation stays, and inpatient discharge (yes or no). Due to differences in data availability for Medicare Advantage enrollees, ED use was only observed for traditional Medicare enrollees. Any inpatient discharge included Medicare Advantage enrollees starting in 2010. To explore a potential mechanism behind changes in acute services use, we measured the monthly number of unique drugs (according to generic drug name) filled by participants with continuous Medicare Part D coverage throughout the observation window. To our knowledge, medication regimen is unexplored in the context of HCBS, even though HCBS may provide support with medication and disease management.

### Stratifications

To assess whether HCBS was differentially beneficial for individuals with complex conditions, we explored 2 chronic condition subgroups: diabetes and Alzheimer disease and related dementias (ADRD). These conditions are particularly relevant for our sample of older adults in the Southeast, where diabetes prevalence is high.^13^ Older adults with diabetes require more support from unpaid caregivers, especially those with more complex medication regimens involving insulin injections.^14^ Further, diabetes is a risk factor for ADRD,^15^ a condition with high rates of ED use and escalating support needs.^16^ We captured diagnosis of both conditions according to validated claims algorithms by the end of the calendar year prior to HCBS initiation.^17^ Diagnosis information was limited to inpatient claims for Medicare Advantage enrollees and available from all outpatient and inpatient claims for traditional Medicare enrollees.

We designated those with 1915(c) enrollment or claims as waiver participants based on the first month of HCBS use and then stratified by waiver and state plan personal care use. All sample states offered 1915(c) waiver programs and 5 states offered a state plan personal care benefit during our study period (South Carolina, West Virginia, Florida, Arkansas, Louisiana). Since some participants were established Medicaid beneficiaries and others initiated Medicaid coverage concurrent with their HCBS benefits, we also explored whether Medicaid status 6 months prior to HCBS initiation influenced results. In eTable 3 and eFigure 4 in Supplement 1, we include additional stratifications based on self-reported characteristics provided in the SCCS baseline survey (sex, high school degree, race and ethnicity) and age at HCBS initiation. Race and ethnicity categories from the SCCS baseline questionnaire were coded as Black, White, and other or unknown for the purposes of this analysis due to sample size limitations. Other or unknown includes American Indian or Alaska Native (only), Asian or Pacific Islander (only), Hispanic/Latino (any), other racial or ethnic group (only), multiple races (excluding Hispanic or Latino), and refuse or do not know responses.

### Statistical Analysis

Linear regressions with an event study structure estimated the probability of inpatient discharge, probability of ED use, and number of unique drugs filled according to lead and lag indicators representing each month relative to first observed HCBS use. Models included calendar year and individual-level fixed effects. We provide event study plots of the event-month estimates. We also estimated models with pooled preperiod and postperiod event months to provide overall preperiod-to-postperiod estimates and to provide stratified results based on the previously described characteristics. All regressions used a 2-sided *t *test to determine statistical significance and we provide 95% CIs. We estimated marginal effects and cluster SEs at the individual level for all models.

We explored sensitivity to model functional form by reestimating results with calendar month-year fixed effects. We also made several adjustments to the sample. First, we analyzed a subgroup of individuals with 2 years of continuous Medicare enrollment to identify persistence over time, plotting 24 total event months centered around HCBS initiation. Second, we lifted the continuous HCBS use requirement, including individuals with inconsistent HCBS use. Third, we limited the sample to individuals who initiated HCBS prior to their respective state’s transition from MAX to TAF. Fourth, we re-estimated baseline models after sequentially dropping each state from the sample. Fifth, we excluded individuals with nursing home use at any point in the observed window to ensure that nursing home admission or discharge did not affect observed changes. Last, we excluded Medicare Advantage enrollees entirely to check that incomplete reporting did not affect results.

## Results

### Sample Characteristics

In a sample of 1218 new HCBS users, 75% were female (911), 77% were Black (938), 19% were White (234), 4% were of other or unknown race or ethnicity (46), and 56% had less than a high school degree (678) (Table 1; eTable 1 in Supplement 1). The mean (SD) age at HCBS initiation was 70.5 (7.5) years (Table 1; eFigure 2 in Supplement 1). Approximately 14% had ADRD and 56% had a diabetes diagnosis in the year prior to initiating HCBS. Seventy-one percent were enrolled in traditional Medicare vs 29% in Medicare Advantage. Almost all (96%) had Medicare Part D coverage for the duration of the study period. Fifty-eight percent had full Medicaid prior to HCBS initiation and 85% received HCBS through a 1915(c) waiver. Twelve percent had a nursing home stay in the pre-HCBS period and 8% had a stay in the post-HCBS period.

**Table 1.** Sample Summary Statistics^a^

Characteristic	No. (%)
Unique individuals	1218 (100.0)
Sex	
Male	307 (25)
Female	911 (75)
Age	
<70 y	647 (53)
>70 y	571 (47)
Age at initiation, mean (SD), y	70.5 (7.5)
Race and ethnicity^b^	
Black	938 (77)
White	234 (19)
Other and unknown^c^	46 (4)
Educational attainment	
Less than HS degree	678 (56)
HS degree or higher	505 (42)
Missing	35 (3)
ADRD diagnosis pre-HCBS	172 (14)
Diabetes diagnosis pre-HCBS	685 (56)
NH stay pre-HCBS	147 (12)
NH stay post-HCBS	97 (8)
Medicare enrollment type	
Medicare Advantage	358 (29)
Traditional Medicare	860 (71)
Medicaid coverage 6 mo prior to HCBS	
No or partial Medicaid	510 (42)
Full Medicaid	708 (58)
Continuous Medicare Part D coverage	
No	47 (4)
Yes	1171 (96)
HCBS through waiver	
State plan	183 (15)
Waiver	1035 (85)
Preperiod outcomes, mean (SD)	
Any emergency department use, %	10.8 (31.1)
Any inpatient discharge, %	7.8 (26.9)
Unique drugs filled	5.7 (3.9)

Abbreviations: ADRD, Alzheimer disease and related dementias; HCBS, home and community-based services; HS, high school; NH, nursing home.

^a^ Variables were measured at the person-month level, with all 1218 sampled individuals contributing 12 person-months. HCBS through waiver indicator is determined in the month of HCBS initiation. Preperiod outcomes of unique drug fills, any emergency department use, and any inpatient discharge are measured across the 6 baseline event months. Unique drugs filled was captured for individuals with Medicare Part D coverage for the entire observation window. The proportion of person-months with an emergency department visit captures traditional Medicare users only. Any emergency department use captures traditional Medicare users only. Any inpatient discharge includes traditional Medicare beneficiaries in all years and Medicare Advantage beneficiaries beginning in 2010.

^b^ Derived by patient self-report. Race and ethnicity categories from the Southern Community Cohort Study^11^ baseline questionnaire were coded as Black, White, and other or unknown for the purposes of this analysis due to sample size limitations.

^c^ Other or unknown includes American Indian or Alaska Native (only), Asian or Pacific Islander (only), Hispanic/Latino (any), other racial or ethnic group (only), multiple races (excluding Hispanic or Latino), and refuse or do not know responses.

### Outcomes

**Figure 1.**
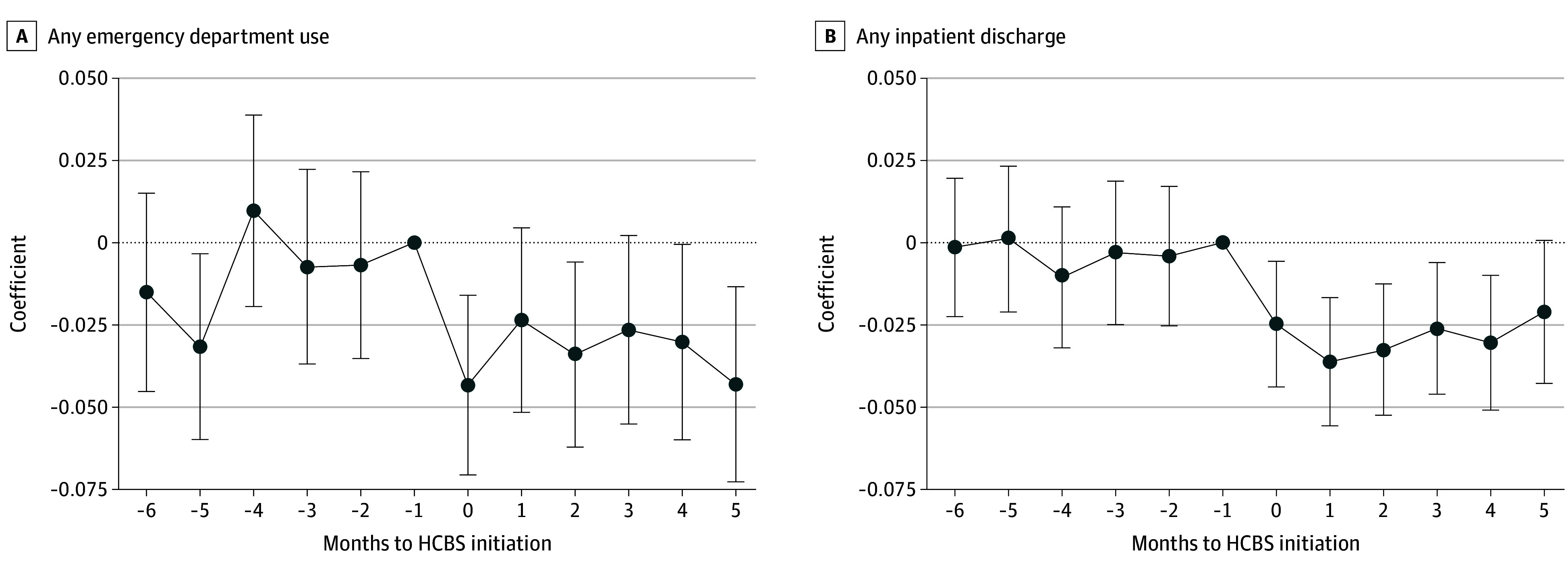
Event Study Dot Plots Showing Dynamic Change in Outcomes Associated With Medicaid Home and Community-Based Services (HCBS) Initiation Estimates were produced using linear regression models with person fixed effects, year fixed effects, and robust SEs. The x-axis represents time relative to HCBS initiation in person-months, where 0 is the first month in which an HCBS flag is detected. Medicare Advantage enrollees are included in the any inpatient discharge variable starting in 2010. Any emergency department use includes observation stays and outpatient admissions for traditional Medicare enrollees. Whiskers indicate 95% CIs.

Unadjusted outcomes in the pre-HCBS period are presented in Table 1. The probability (SD) of having any ED use was 10.8% (31.1%) and any inpatient discharge was 7.8% (26.9%). The mean (SD) number of unique drugs filled was 5.7 (3.9). Transitioning from pre– to post–HCBS initiation was associated with a decrease in the probability of having any ED use (−2.70 percentage points; 95% CI, −4.18 to −1.22 percentage points) (Table 2) and a decrease in the probability of any inpatient discharge (−2.63 percentage points; 95% CI, −3.75 to −1.51 percentage points). These differences represent a 24% decrease in the within-person probability of any ED use and a 32% decrease in inpatient discharge down from adjusted pre-HCBS probabilities of 11.4% and 8.1%. Event study estimates were largely not significantly different from 0 in the months prior to HCBS initiation followed by a sharp decrease in the first month of HCBS use that persisted for the entire postperiod (Figure 1).

**Table 2.** Decrease in Acute Services Use Outcomes Associated With Medicaid HCBS Initiation Stratified by Health Services’ Use Subcategories^a^

Subcategory	No. of observations	No. of unique individuals	Adjusted pre-HCBS mean	Marginal effect (95% CI)	*P* value
**Any emergency department use**					
Full sample	10 320	860	11.38	–2.70 (–4.18 to –1.22)	<.001
Diabetes diagnosis	6408	534	12.06	–2.51 (–4.46 to –0.57)	.01
ADRD diagnosis	1680	140	13.56	–2.71 (–6.85 to 1.43)	.20
Waiver user	8496	708	11.39	–2.11 (–3.75 to –0.47)	.01
State plan user	1824	152	11.34	–5.46 (–8.94 to –1.97)	.003
Full Medicaid	6048	504	11.83	–3.65 (–5.62 to –1.68)	<.001
Partial or no Medicaid	4272	356	10.75	–1.37 (–3.62 to 0.89)	.24
**Any inpatient discharge**					
Full sample	14 064	1172	8.11	–2.63 (–3.75 to –1.51)	<.001
Diabetes diagnosis	7968	664	9.73	–3.33 (–4.85 to –1.81)	<.001
ADRD diagnosis	2028	169	11.37	–4.20 (–7.73 to –0.68)	.02
Waiver user	11 964	997	8.45	–2.69 (–3.93 to –1.44)	<.001
State plan user	2100	175	6.22	–2.35 (–4.86 to 0.16)	.07
Full Medicaid	8184	682	7.21	–1.83 (–3.22 to –0.44)	.01
Partial or no Medicaid	5880	490	9.37	–3.74 (–5.60 to –1.89)	<.001

Abbreviations: ADRD, Alzheimer disease and related dementias; HCBS, home- and community-based services.

^a^ Estimates were produced using pooled pre and post outcomes with person fixed effects, year fixed effects, and robust SEs. Both variables are observed at the person-month level. Medicare Advantage enrollees are included in the any inpatient discharge variable starting in 2010. Any emergency department use includes observation stays and outpatient admissions for traditional Medicare enrollees only. Medicaid coverage (full Medicaid and partial or none) is based on Medicaid coverage level 6 months prior to HCBS initiation. Waiver and state plan use indicators are determined in the month of HCBS initiation. Estimates are presented as percentages.

Initiating HCBS was associated with 0.31 (95% CI, 0.20-0.43) additional drugs filled per month, representing a 5.5% change (eTable 2 in Supplement 1). However, event study results suggest that the number of drugs filled was already increasing prior to HCBS initiation and continued to increase thereafter, with no clear discontinuity beginning with HCBS use (eFigure 3 in Supplement 1). Given the ambiguity of these drug outcomes, we explored stratified results for inpatient discharge and ED service use outcomes only.

### Stratifications

**Figure 2.**
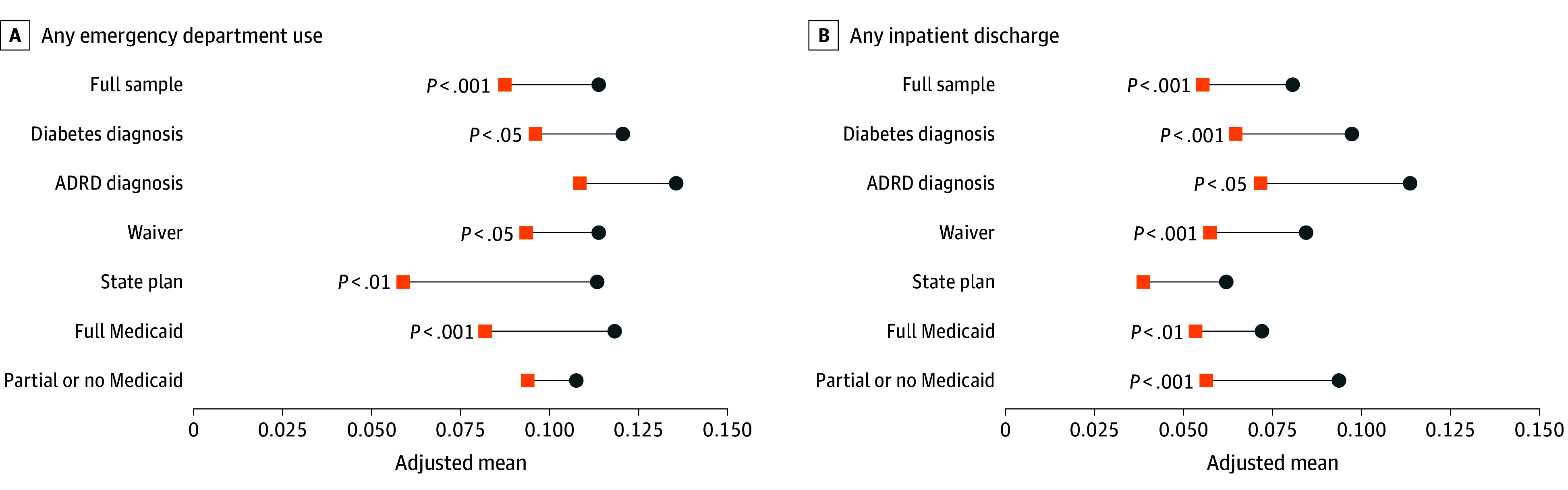
Dot Plots Showing Decrease in Acute Services Use Outcomes Associated With Medicaid Home and Community-Based Services (HCBS) Initiation Stratified by Health Services’ Use Subcategories Marginal effects (connecting line) of initiating HCBS with person fixed effects, year fixed effects, and robust SEs are shown. The blue circles represent adjusted means in the preperiod and orange squares represent the postperiod adjusted means. Both outcomes are observed at the person-month level. Medicare Advantage enrollees are included in the any inpatient discharge variable starting in 2010. Any emergency department use includes observation stays and outpatient admissions for traditional Medicare enrollees only. Time-invariant Medicaid coverage (full Medicaid and partial or none) is based on person-month −6 relative to HCBS initiation. Time-invariant waiver and state plan use indicators are determined in the month of HCBS initiation. ADRD indicates Alzheimer disease and related dementias.

All subgroups showed reduced ED use and inpatient discharges, including subgroups based on comorbidity, insurance coverage, and demographic characteristics (Table 2, Figure 2; eTable 3 and eFigure 4 in Supplement 1). State plan and full Medicaid users experienced larger decreases in ED use than the broader sample. State plan users experienced a decrease of 5.46 percentage points (95% CI, –8.94 to –1.97 percentage points) in the probability of ED use and full Medicaid users experienced a decrease of 3.65 (95% CI, –5.62 to –1.68). Inpatient discharges decreased more for participants with complex conditions, as the probability of inpatient discharge decreased by 3.33 (95% CI, −4.85, −1.81) percentage points for persons with diabetes and 4.20 (95% CI, −7.73, −0.68) for those with ADRD. Further, reduced inpatient discharge was concentrated among those with partial or no Medicaid prior to initiating HCBS (−3.74 percentage points; 95% CI, −5.60 to −1.89 percentage points). Individuals initiating HCBS before age 65 years had more pronounced decreases in ED use than older age groups but similar changes in inpatient discharges.

### Sensitivity Analyses

Adjustments to the model’s functional form and sample suggest results consistent with the main analysis (eTables 4 and 5, eFigures 5-11 in Supplement 1). Reductions in inpatient discharge and ED use persisted 12 months after HCBS initiation (eFigure 5 in Supplement 1). After limiting to participants with HCBS use observed in MAX data (excluding TAF years), the ED reductions were attenuated but inpatient results were consistent with the main analysis (eFigure 7 in Supplement 1). Results were maintained after excluding participants with nursing home use and Medicare Advantage enrollees (eFigures 8 and 9 in Supplement 1). The sequential exclusion of sample states did not indicate that results were concentrated in 1 state. Event-month estimates were less precise after excluding Mississippi, which comprised a large share of our sample (eFigure 11 in Supplement 1).

## Discussion

In a cohort of low-income southeastern older adults, the within-person probability of any ED use and inpatient discharge decreased significantly starting in the first month of Medicaid HCBS use. The observed decrease lasted for 6 months following HCBS initiation, and sensitivity analyses indicate reductions continued for at least 1 year. All subgroup analyses showed a negative association between HCBS initiation and acute services use. Participants with ADRD and diabetes had the largest decreases in inpatient discharges, suggesting that participants with these complex conditions may experience additional benefits. Inpatient discharge decreases were also more pronounced among individuals with no or partial Medicaid prior to HCBS use.

Our findings extend the prior literature showing higher rates of avoidable hospitalizations in HCBS relative to nursing homes. For example, HCBS users have more avoidable hospitalizations than nursing home residents,^18,19^ as do individuals transitioning out of nursing homes to HCBS.^20^ This article does not contradict these findings but instead addresses a distinct research question that explores the potential value of Medicaid HCBS relative to the absence of Medicaid-funded LTSS. That is, we explored changes when new users transition from no Medicaid LTSS support to formal Medicaid-funded HCBS as opposed to conducting a cross-sectional analysis of HCBS vs nursing home care.

To our knowledge, only 2 studies have followed acute services use among new HCBS users over time. They compared HCBS with nursing home users with mixed results. A 2008 analysis of Indiana’s 1915(c) waiver included 174 HCBS users and found that their costs post–HCBS initiation were lower than those for nursing home users, but also with faster growth in inpatient care and associated costs.^21^ The second study found that, among new LTSS recipients in California’s Medi-Cal program, HCBS users had lower acute care costs over the first 12 months of use compared with nursing home users.^22^ Our analysis builds on these studies, using a broader multistate sample to observe trends before and after initiating HCBS and to explore heterogeneity across different populations.

We identify important differences in reduced inpatient discharge and ED use across subgroups that are worth noting as states work to reduce unmet need and improve HCBS quality. Reflecting our study setting, approximately half of sample participants had diabetes and had higher hospitalization rates pre-HCBS and larger post-HCBS decreases in inpatient discharges than the broader sample. Those with ADRD also experienced large inpatient discharge reductions, suggesting there may be more potential for HCBS to improve outcomes for those with complex care needs.

Despite prior evidence that more intensive HCBS service use is associated with reduced risk of hospitalizations and nursing home admission,^6,23^ we found mixed results when comparing individuals using waiver and state plan personal care services. Consistent with waiver programs providing a broader scope of services, individuals who initiated HCBS as a waiver participant had significant decreases in inpatient use while state plan users did not. However, state plan users experienced larger decreases in ED use than waiver users, perhaps because state plan services may be more readily available than waiver programs. Results also differed based on individuals’ Medicaid participation prior to HCBS initiation. Those who were already enrolled in full Medicaid had larger decreases in ED use but smaller decreases in inpatient use. Better measurement of HCBS processes and type of services provided may assist in the understanding of the mechanisms behind this observed heterogeneity.

Our medication analyses did not reveal clear changes in the number of medications filled coinciding with HCBS initiation, although we observed increased medication use leading up to HCBS initiation and continuing thereafter. This trend suggests the challenges that older adults living in the community may face as they juggle complex medication regimens. A survey of Medicaid HCBS users identified that 18% of respondents ages 65 to 74 years needed assistance with taking medications, highlighting the potentially critical role of HCBS in medication use for older adults.^24^ Claims-based fills may not capture the aspect of medication use through which HCBS is most impactful. More research is needed to assess how HCBS may improve medication management and reduce avoidable acute services use.

### Limitations

This analysis has important limitations. First, our study design is not causal; services utilized concurrently with HCBS or newfound diagnoses could influence our outcome measures. We cannot identify non-Medicaid sources of support that individuals may have been using prior to Medicaid HCBS initiation or concurrently. Second, the Centers for Medicare & Medicaid Services introduced a new Medicaid data format, TAF, during the study period, which may affect measurement of HCBS. Our sensitivity analyses suggest that results remain largely consistent when studying only pretransition data, although the sample size is limiting. Third, we have only inpatient diagnosis information for Medicare Advantage enrollees starting in 2010. Fourth, 23% of our sample enrolled in full Medicaid for the first time in the same month as HCBS initiation. Gaining Medicaid assistance with Medicare out-of-pocket costs could also influence study outcomes. However, we still observed decreases in ED use and inpatient discharges among new HCBS users with full Medicaid prior to HCBS. Last, we studied a unique sample of predominantly Black and low-income older adults in the Southeast and our study period ends in 2018; therefore, the generalizability of our findings may be limited.

## Conclusions

This analysis of a large sample of older adults with low income suggests that initiation of Medicaid HCBS was associated with decreases in inpatient discharges and ED use. Future research should explore additional outcomes that better inform policies that can improve HCBS outcomes and our understanding of its tradeoffs with acute services use.

## Notes

Supplement 1. **eMethods. **Identification of HCBS Use in MAX and TAF Data **eFigure 1.** Consort Diagram **eTable 1.** Distribution of Cohort by State, Year, and Month of HCBS Initiation **eFigure 2.** Distribution of Cohort Age at Month of HCBS Initiation **eTable 2.** Pre-Post Change in Unique Drug Fills Associated with HCBS Initiation **eFigure 3.** Plotted Change in Unique Drug Fills Associated with HCBS Initiation **eTable 3.** Decrease in Acute Service Use Outcomes Associated with HCBS Initiation Across Demographic Groups **eFigure 4.** Plotted Decrease in Acute Service Use Outcomes Across Demographic Groups **eTable 4.** Pre-Post Change in Outcomes Associated with HCBS Initiation with Sample Adjustments **eFigure 5.** Dynamic Change in Outcomes Associated with HCBS Initiation and Extended Study Period **eFigure 6.** Dynamic Change in Outcomes Associated with HCBS Initiation without HCBS Continuous Use Requirement **eFigure 7.** Dynamic Change in Outcomes Associated with HCBS Initiation Among Pre-TAF Sample **eFigure 8.** Dynamic Change in Outcomes Associated with HCBS Initiation when Excluding Participants with Any Nursing Home Use **eFigure 9.** Dynamic Change in Outcomes Associated with HCBS Initiation when Excluding Medicare Advantage Enrollees **eTable 5.** Pre-Post Change in Outcomes Associated with HCBS Initiation with Model Adjustments **eFigure 10.** Dynamic Change in Outcomes Associated with HCBS Initiation with Month-Year Fixed Effects **eFigure 11.** Dynamic Change in Outcomes Resulting from State Leave One Out Models

Supplement 2.
Data Sharing Statement
