# Supplementary material for: Medicaid Home and Community-Based Services Initiation and Acute Services Use
Source: JAMA Health Forum. 2026 Mar 27;7(3):e260206. doi: 10.1001/jamahealthforum.2026.0206 (PMC13032151; doi:10.1001/jamahealthforum.2026.0206)
Supplement: Supplement 1. — eMethods. Identification of HCBS Use in MAX and TAF Data eFigure 1. Consort Diagram eTable 1. Distribution of Cohort by State, Year, and Month of HCBS Initiation eFigure 2. Distribution of Cohort Age at Month of HCBS Initiation eTable 2. Pre-Post Change in Unique Drug Fills Associated with HCBS Initiation eFigure 3. Plotted Change in Unique Drug Fills Associated with HCBS Initiation eTable 3. Decrease in Acute Service Use Outcomes Associated with HCBS Initiation Across Demographic Groups eFigure 4. Plotted Decrease in Acute Service Use Outcomes Across Demographic Groups eTable 4. Pre-Post Change in Outcomes Associated with HCBS Initiation with Sample Adjustments eFigure 5. Dynamic Change in Outcomes Associated with HCBS Initiation and Extended Study Period eFigure 6. Dynamic Change in Outcomes Associated with HCBS Initiation without HCBS Continuous Use Requirement eFigure 7. Dynamic Change in Outcomes Associated with HCBS Initiation Among Pre-TAF Sample eFigure 8. Dynamic Change in Outcomes Associated with HCBS Initiation when Excluding Participants with Any Nursing Home Use eFigure 9. Dynamic Change in Outcomes Associated with HCBS Initiation when Excluding Medicare Advantage Enrollees eTable 5. Pre-Post Change in Outcomes Associated with HCBS Initiation with Model Adjustments eFigure 10. Dynamic Change in Outcomes Associated with HCBS Initiation with Month-Year Fixed Effects eFigure 11. Dynamic Change in Outcomes Resulting from State Leave One Out Models [file jamahealthforum-e260206-s001.pdf]

Supplemental Online Content

Keesee E, Fabius CD, Kim J, Stevenson D, Keohane LM. Medicaid home and community-based services initiation and acute services use. *JAMA Health Forum*. 2026;7(3):e260206. doi:10.1001/jamahealthforum.2026.0206

eMethods. Identification of HCBS Use in MAX and TAF Data

eFigure 1. Consort Diagram

eTable 1. Distribution of Cohort by State, Year, and Month of HCBS Initiation

eFigure 2. Distribution of Cohort Age at Month of HCBS Initiation

eTable 2. Pre-Post Change in Unique Drug Fills Associated with HCBS Initiation

eFigure 3. Plotted Change in Unique Drug Fills Associated with HCBS Initiation

eTable 3. Decrease in Acute Service Use Outcomes Associated with HCBS Initiation Across Demographic Groups

eFigure 4. Plotted Decrease in Acute Service Use Outcomes Across Demographic Groups

eTable 4. Pre-Post Change in Outcomes Associated with HCBS Initiation with Sample Adjustments

eFigure 5. Dynamic Change in Outcomes Associated with HCBS Initiation and Extended Study Period

eFigure 6. Dynamic Change in Outcomes Associated with HCBS Initiation without HCBS Continuous Use Requirement

eFigure 7. Dynamic Change in Outcomes Associated with HCBS Initiation Among Pre-TAF Sample

eFigure 8. Dynamic Change in Outcomes Associated with HCBS Initiation when Excluding Participants with Any Nursing Home Use

eFigure 9. Dynamic Change in Outcomes Associated with HCBS Initiation when Excluding Medicare Advantage Enrollees

eTable 5. Pre-Post Change in Outcomes Associated with HCBS Initiation with Model Adjustments

eFigure 10. Dynamic Change in Outcomes Associated with HCBS Initiation with Month-Year Fixed Effects

eFigure 11. Dynamic Change in Outcomes Resulting from State Leave One Out Models

This supplemental material has been provided by the authors to give readers additional information about their work.

© 2026 Keesee E et al. *JAMA Health Forum*.

eMethods

Identification of HCBS Use in MAX and TAF Data

Medicaid MAX Personal Summary File<br>Medicaid MAX Other Services	Medicaid TAF Demographic and Eligibility Base File<br>Medicaid TAF Other Services
**1915(c) waiver participation**	
Meets any of these 2 conditions:<br><br>1. Enrolled in a 1915(c) waiver program:<br><br>MAX_WAIVER_TYPE_**_MO_** values:<br><br>G, H, I, J, K, L, M, N, O, P<br><br>2. Has an Other Services claim covered by a 1915(c) waiver program:<br><br>CLTC_FLAG values:<br>30, 31, 32, 33, 34, 35, 36, 37, 38, 39, 40	Meets any of these 2 conditions:<br><br>1: Enrolled in a 1915(c) waiver program:<br><br>WVY_TYPE_CD_**_** values:<br><br>06, 07, 08, 09, 10, 11, 12, 13, 14, 15, 16, 17, 18, 19, 20, 33<br><br>2: Has an Other Services claim covered by a 1915(c) waiver program as indicated by meeting any of these 3 conditions:<br><br>a: PGM_TYPE_CD values:<br><br>07<br><br>b: WVY_TYPE_CD values:<br><br>06, 07, 08, 09, 10, 11, 12, 13, 14, 15, 16, 17, 18, 19, 20, 33<br><br>c: HCBS_SRVC_CD values:<br><br>04<br><br>*We used modified approaches to measuring HCBS users in these states due to data issues:<br><br>North Carolina (2014 onwards), South Carolina (2014 onwards), Alabama (10/2018 onwards):<br><br>Has an Other Services claim covered by a 1915(c) waiver program<br><br>Virginia (2015 onwards):<br><br>Enrolled in a 1915(c) waiver program

© 2026 Keesee E et al. *JAMA Health Forum*.

	Arkansas (2016 onwards) and Florida (2017 onwards) excluded for questionable accuracy of HCBS data relative to trends in previous years.
**Personal care claim - has an Other Services claim for personal care services:**	
PRCDR_CD values:<br><br>T1019, T1020, 99509, S5125, S5126	LINE_PRCDR_CD values:<br><br>T1019, T1020, 99509, S5125, S5126

*Methodology for identifying 1915(c) waiver use and personal care claims based on Stepanczuk, Cara, Caitlin Murray, Alexandra Carpenter, and Andrea Wysocki; "Methodology for Identifying Medicaid Long-Term Services and Supports Expenditures and Users, 2022;" Mathematica, August 29, 2024

Identification of HCBS Users in Medicaid Claims by State and Type of HCBS Flag

	Waiver Claims	Waiver Enrollment	Personal Care Only	1st TAF Year
**Alabama***	01/2006-12/2018	01/2006-09/2018	01/2006-12/2018	2014
**Arkansas***	01/2006-12/2015	01/2006-12/2015	01/2006-12/2015	2016
**North Carolina***	01/2006-12/2018	01/2006-12/2013	01/2006-12/2018	2014
**South Carolina***	01/2006-12/2018	01/2006-12/2013	01/2006-12/2018	2015
**Florida***	01/2006-12/2016	01/2006-12/2016	01/2006-12/2016	2014
**Georgia**	01/2006-12/2018	01/2006-12/2018	01/2006-12/2018	2016
**West Virginia**	01/2006-12/2018	01/2006-12/2018	01/2006-12/2018	2016
**Louisiana**	01/2006-12/2018	01/2006-12/2018	01/2006-12/2018	2016
**Mississippi**	01/2006-12/2018	01/2006-12/2018	01/2006-12/2018	2016
**Kentucky**	01/2006-12/2018	01/2006-12/2018	01/2006-12/2018	2015
**Virginia***	01/2006-12/2014	01/2006-12/2018	01/2006-12/2018	2015

NOTE: Summarizes state-specific changes noted above. Years in which a given indicator of HCBS (waiver claim, enrollment, personal care) was used varied across states based on stability of data and author knowledge of policy changes (such as MAX to TAF transition timing). *States with altered strategy for HCBS identification due to improbable changes in Medicaid HCBS use, largely coinciding with MAX-TAF transition. HCBS = home- and community-based services.

© 2026 Keesee E et al. *JAMA Health Forum*.

eFigure 1. Consort Diagram

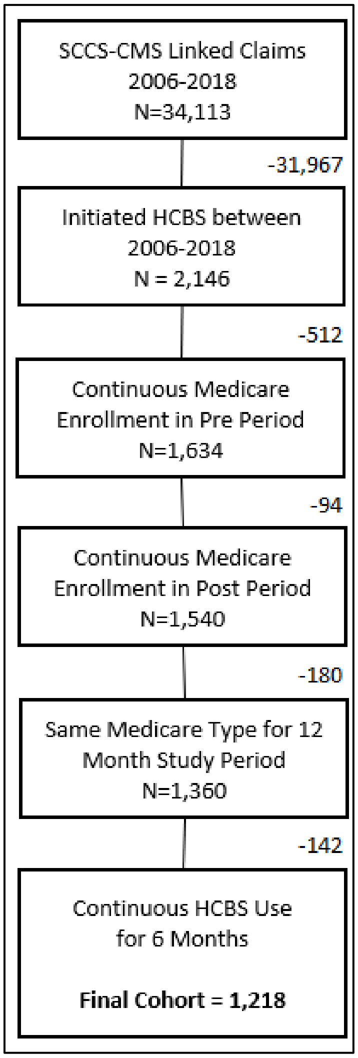

NOTE: HCBS = home- and community-based services

© 2026 Keesee E et al. *JAMA Health Forum*.

eTable 1. Distribution of Cohort by State, Year, and Month of HCBS Initiation

A. Unique Individuals by State of Residence

	N	%
Alabama	107	8.78
Arkansas	109	8.95
Florida	52	4.27
Georgia	130	10.67
Kentucky	36	2.96
Louisiana	69	5.67
Mississippi	445	36.54
North Carolina	31	2.55
South Carolina	112	9.2
Virginia	70	5.75
West Virginia	57	4.68
Total	1218	100

B. Unique Individuals by Year of HCBS Initiation

	N	%
2006, 2007	77	6.32
2008	60	4.93
2009	73	5.99
2010	130	10.67
2011	124	10.18
2012	105	8.62
2013	134	11
2014	162	13.3
2015	119	9.77
2016	84	6.9
2017	101	8.29
2018	49	4.02
Total	1,218	100.00

C. Unique Individuals by Month of HCBS Initiation

	N	%
January	125	10.26
February	90	7.39
March	105	8.62
April	121	9.93
May	98	8.05
June	97	7.96
July	112	9.2
August	90	7.39
September	104	8.54
October	110	9.03
November	90	7.39
December	76	6.24
Total	1,218	100.00

NOTE: HCBS = home- and community-based services.

© 2026 Keesee E et al. *JAMA Health Forum*.

eFigure 2. Distribution of Cohort of Age at Month of HCBS Initiation

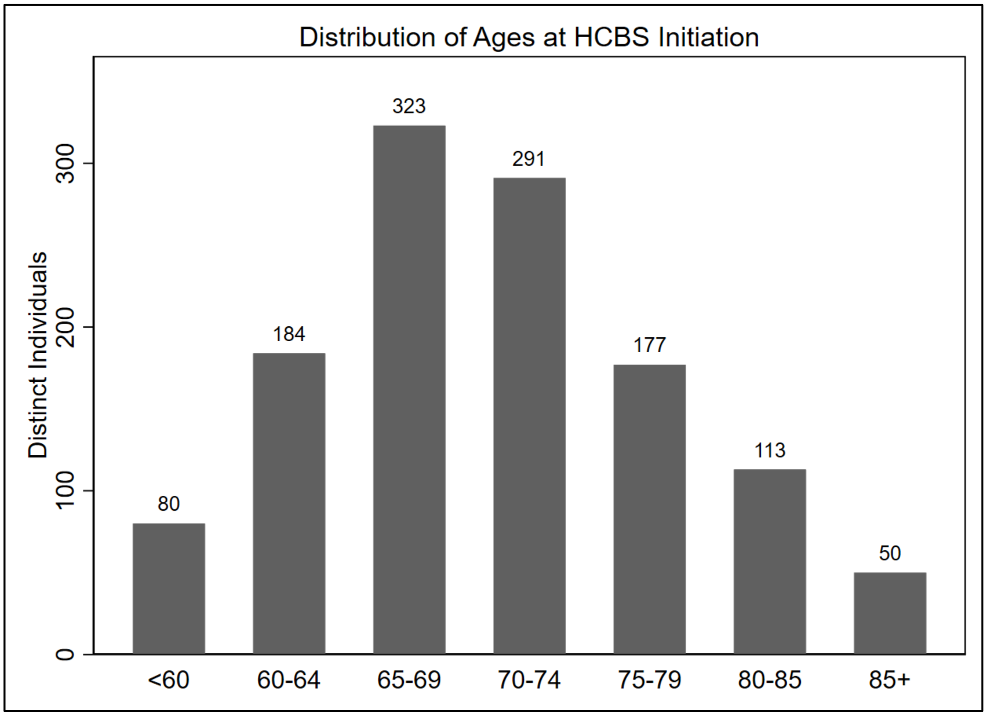

NOTE: HCBS = home- and community-based services.

© 2026 Keesee E et al. *JAMA Health Forum*.

eTable 2. Pre-Post Change in Unique Drug Fills Associated with HCBS Initiation

	Unique Drugs Filled
Adjusted Pre-HCBS Mean	5.591
Marginal Effect	0.313***
[95% CI]	[0.198, 0.428]
Observations	14052
Unique Individuals	1171
Traditional Medicare Enrollees	X
Medicare Advantage Enrollees	X
Continuous Part D Coverage	X

NOTE: ***p<0.001, **p<0.01, *p<0.05. Estimates were produced using pooled pre and post outcomes with person fixed-effects, year fixed-effects, and robust standard errors. Unique drug fills include individuals with Part D for the entire observation window. HCBS = home- and community-based services. ME = marginal effect. CI = confidence interval.

© 2026 Keesee E et al. *JAMA Health Forum*.

eFigure 3. Plotted Change in Drug Fills per Participant Associated with HCBS Initiation

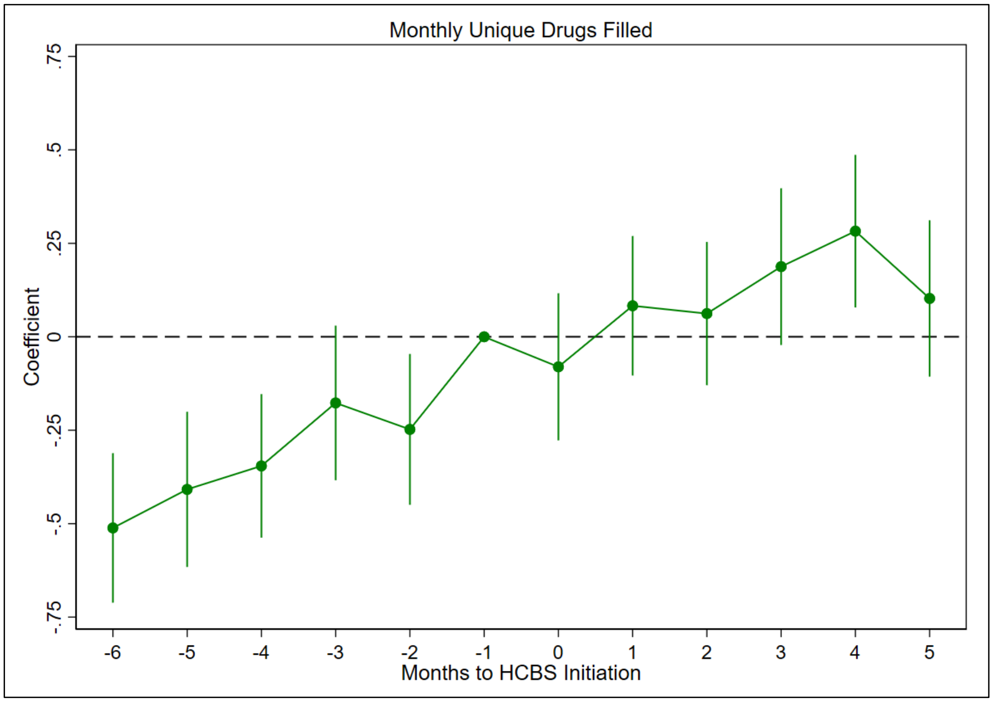

NOTE: Estimates were produced using linear regression models with person-fixed effects, year fixed effects, and robust standard errors. The X axis represents time relative to HCBS initiation in person-months, where 0 is the first month in which an HCBS flag is detected. Unique drug fills include individuals with continuous Part D coverage pre- and post-HCBS.

© 2026 Keesee E et al. *JAMA Health Forum*.

eTable 3. Decrease in Acute Service Use Outcomes Associated with HCBS Initiation Across Demographic Groups

Any Emergency Department Use					
	Observations	Unique Individuals	Adjusted Pre-HCBS Mean	Marginal Effect	[95% CI]
Full Sample	10320	860	0.114	-0.027***	-[0.042, -0.012]
Initiation Ages <65	2388	199	0.127	-0.053**	-[0.084, -0.023]
Initiation Ages 65-74	5172	431	0.108	-0.012	-[0.033, 0.009]
Initiation Ages 75+	2760	230	0.113	-0.032*	-[0.061, -0.002]
<HS Degree	5916	493	0.108	-0.033**	-[0.054, -0.013]
HS Degree	4056	338	0.120	-0.019	-[0.041, 0.003]
Female	7548	629	0.116	-0.020*	-[0.037, -0.003]
Male	2772	231	0.108	-0.046**	-[0.076, -0.016]
White/Other	2472	206	0.114	-0.036*	-[0.067, -0.006]
Black	7848	654	0.114	-0.024**	-[0.041, -0.007]
**Any Inpatient Discharge**					
	Observations	Unique Individuals	Adjusted Pre-HCBS Mean	Marginal Effect	[95% CI]
Full Sample	14064	1172	0.081	-0.026***	-[0.038, -0.015]
Age <65	3012	251	0.069	-0.021	-[0.047, 0.005]
Ages 65-74	7128	594	0.085	-0.031***	-[0.047, -0.016]
Ages 75+	3924	327	0.083	-0.022*	-[0.042, -0.001]
<HS Degree	7824	652	0.079	-0.026***	-[0.040, -0.012]
HS Degree	5820	485	0.086	-0.031**	-[0.050, -0.012]
Female	10476	873	0.080	-0.027***	-[0.040, -0.014]
Male	3588	299	0.085	-0.025*	-[0.049, 0.000]
White/Other	3264	272	0.086	-0.027	-[0.054, 0.001]
Black	10800	900	0.080	-0.026***	-[0.038, -0.014]

NOTE: ***p<0.001, **p<0.01, *<0.05. Estimates were produced using pooled pre and post outcomes with person-fixed effects, year fixed effects, and robust standard errors. Both variables are observed at the person-month level. Medicare Advantage enrollees are included in the any inpatient discharge variable starting in 2010. Any emergency department use includes observation stays and outpatient admissions for traditional Medicare enrollees only. Age is measured at HCBS initiation. All other demographic characteristics are pulled from the SCCS baseline survey. 'Other and Unknown' category of race includes self-reported: Hispanic/Latino, Asian or Pacific Islander, American Indian or Alaska Native, Other racial or ethnic group, Mixed Race, 'refuse,' and 'don't know' responses. HCBS = home - and community-based services.

© 2026 Keesee E et al. *JAMA Health Forum*.

eFigure 4. Plotted Decrease in Acute Service Use Outcomes Across Demographic Groups

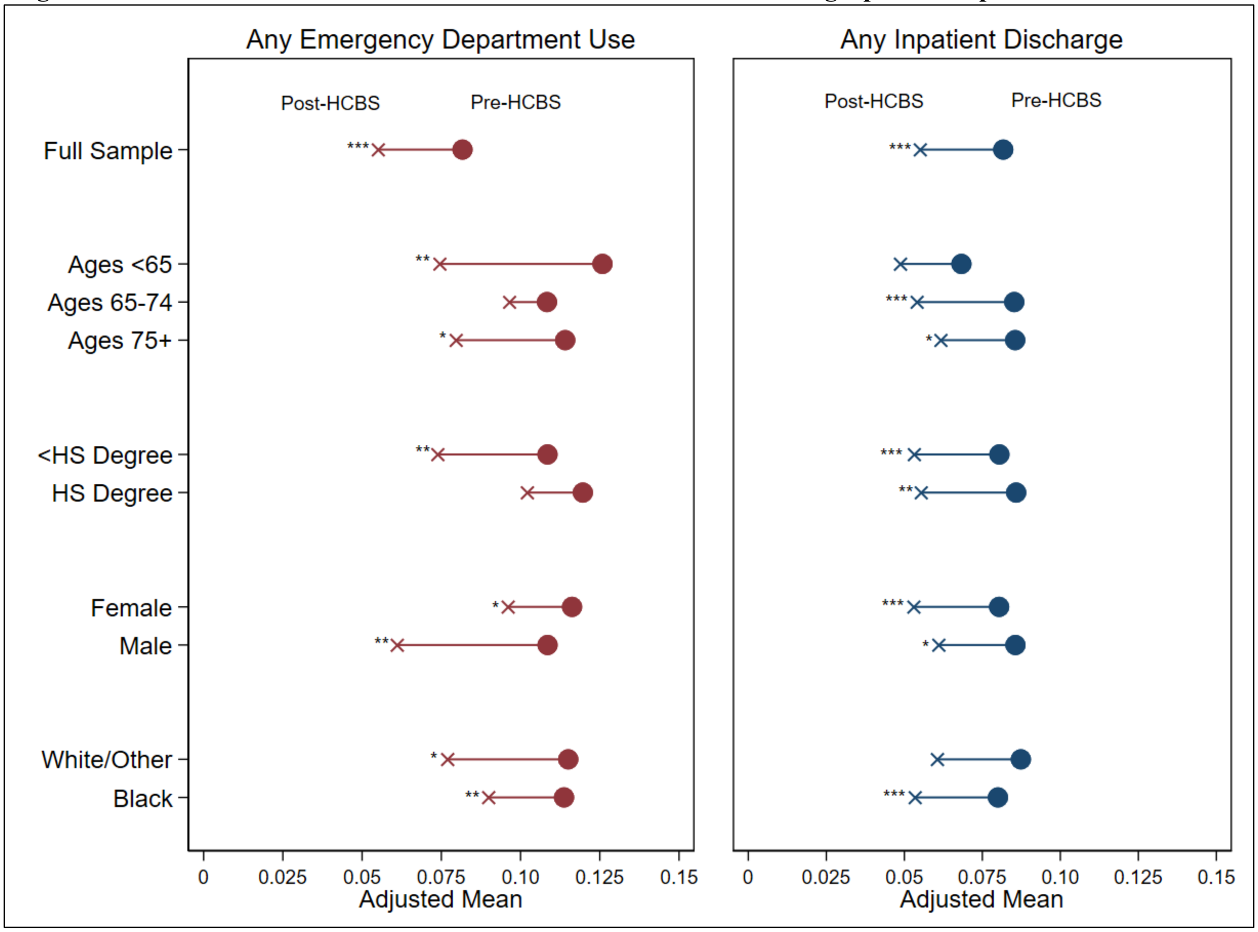

NOTE: ***p<0.001, **p<0.01, *<0.05. Figure shows marginal effect (connecting line) of initiating HCBS with person-fixed effects, year fixed effects, and robust standard errors. The solid dots represent adjusted mean of the outcome in the pooled pre-period and 'X's represent the post-period predictive margins. Both outcomes are observed at the person-month level. Medicare Advantage enrollees are included in the any inpatient discharge variable starting in 2010. Any emergency department use includes observation stays and outpatient admissions for traditional Medicare enrollees only. Age is measured at HCBS initiation. All other demographic characteristics are pulled from the SCCS baseline survey. 'Other and Unknown' category of race includes self-reported: Hispanic/Latino, Asian or Pacific Islander, American Indian or Alaska Native, Other racial or ethnic group, Mixed Race, 'refuse,' and 'don't know' responses. HCBS = home- and community-based services.

© 2026 Keesee E et al. *JAMA Health Forum*.

eTable 4. Pre-Post Change in Outcomes Associated with HCBS Initiation with Sample Adjustments

	Any Emergency Department Use	Any Inpatient Discharge	Unique Drugs Filled
**A. 24 Event Months**			
Adjusted Pre-HCBS Mean	0.109	0.070	5.531
Marginal Effect	-0.024**	-0.024***	0.382***
[95% CI]	-[0.040,	-[0.035,	[0.253,
	-0.009]	-0.012]	0.512]
Observations	15336	20568	20496
Unique Participants	639	857	854
**B. No HCBS Continuous Use Requirement**			
Adjusted Pre-HCBS Mean	0.113	0.083	5.468
Marginal Effect	-0.023**	-0.023***	0.304***
[95% CI]	-[0.037,	-[0.034,	[0.190,
	-0.008]	-0.012]	0.417]
Observations	11484	15588	15896
Unique Participants	957	1299	1341
**C. Pre-TAF Transition**			
Adjusted Pre-HCBS Mean	0.106	0.081	5.641
Marginal Effect	-0.017*	-0.027***	0.355***
[95% CI]	-[0.034,	-[0.041,	[0.217,
	-0.001]	-0.013]	0.492]
Observations	7248	8904	9144
Unique Participants	604	742	762
**D. Nursing Home Users Excluded**			
Adjusted Pre-HCBS Mean	0.111	0.640	5.574
Marginal Effect	-0.028***	-0.021***	0.288***
[95% CI]	[-0.044,	[-0.032,	[0.173,
	-0.013]	-0.010]	0.404]
Observations	8748	11940	12060
Unique Participants	729	995	1005
**E. Medicare Advantage Enrollees Excluded**			
Adjusted Pre-HCBS Mean	0.114	0.088	5.814
Marginal Effect	-0.027***	-0.031	0.302***
[95% CI]	[-0.042,	[-0.045,	[0.158,
	-0.012]	-0.018]	0.446]
Observations	10320	10320	9804
Unique Participants	860	860	817
Traditional Medicare Enrollees	X	X	X
Medicare Advantage Enrollees		X	X
Continuous Part D Coverage			X

NOTE: ***p<0.001, **p<0.01, *p<0.05. Estimates were produced using pooled pre and post outcomes and person fixed effected, year fixed effects, and robust standard errors. Medicare Advantage enrollees are included in the any inpatient discharge variable starting in 2010. Any emergency department use includes observation stays and outpatient admissions. Unique drugs filled include participants with Part D for the entire observation window. Panel A includes individuals with 12 continuous months of Medicare enrollment before and after HCBS initiation. Panel B lifts the continuous HCBS requirement in the post-period,

© 2026 Keesee E et al. *JAMA Health Forum*.

measuring outcomes for across the 6-month post period regardless of consistent HCBS use. Panel C includes individuals that started services before their respective state's transition to TAF. Panel D. excludes individuals with nursing home use at any point in the observation window. Panel E excluded Medicare Advantage enrollees. HCBS = home- and community-based services. CI = confidence interval.

© 2026 Keesee E et al. *JAMA Health Forum*.

eFigure 5. Dynamic Change in Outcomes Associated with HCBS Initiation and Extended Study Period

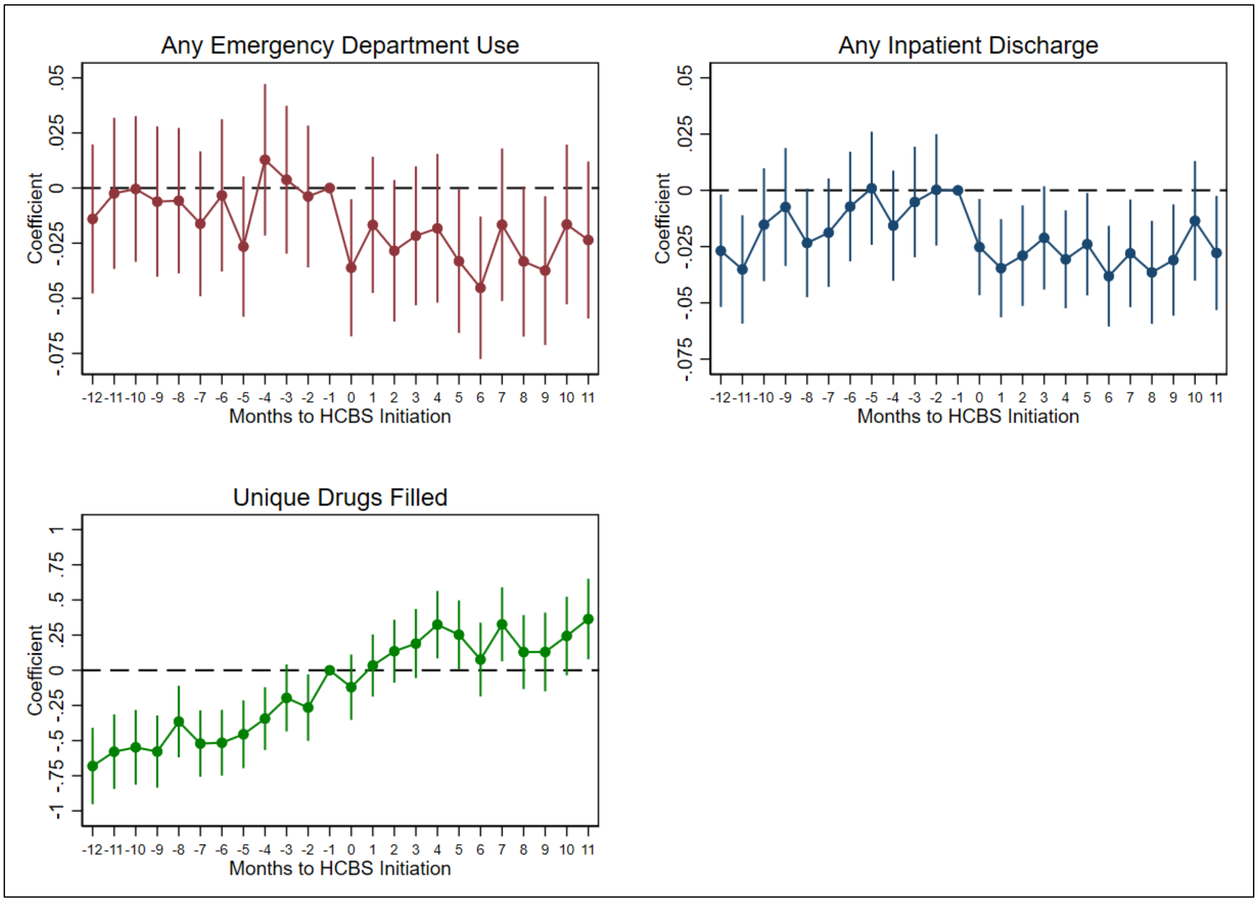

NOTE: Model sample includes individuals with 24 months of continuous Medicare enrollment centered on HCBS initiation. All individuals had 12 months of continuous HCBS use, allowing 1 month gaps. Estimates were produced using linear regression models with person-fixed effects, year fixed effects, and robust standard errors. The X axis represents time relative to HCBS initiation in person-months, where 0 is the first month in which an HCBS flag is detected. Medicare Advantage enrollees are included in the any inpatient discharge variable starting in 2010. Any emergency department use includes observation stays and outpatient admissions. Unique drugs filled include individuals with Part D for the entire observation window. HCBS = home- and community-based services.

© 2026 Keesee E et al. *JAMA Health Forum*.

eFigure 6. Dynamic Change in Outcomes Associated with HCBS Initiation without HCBS Continuous Use Requirement

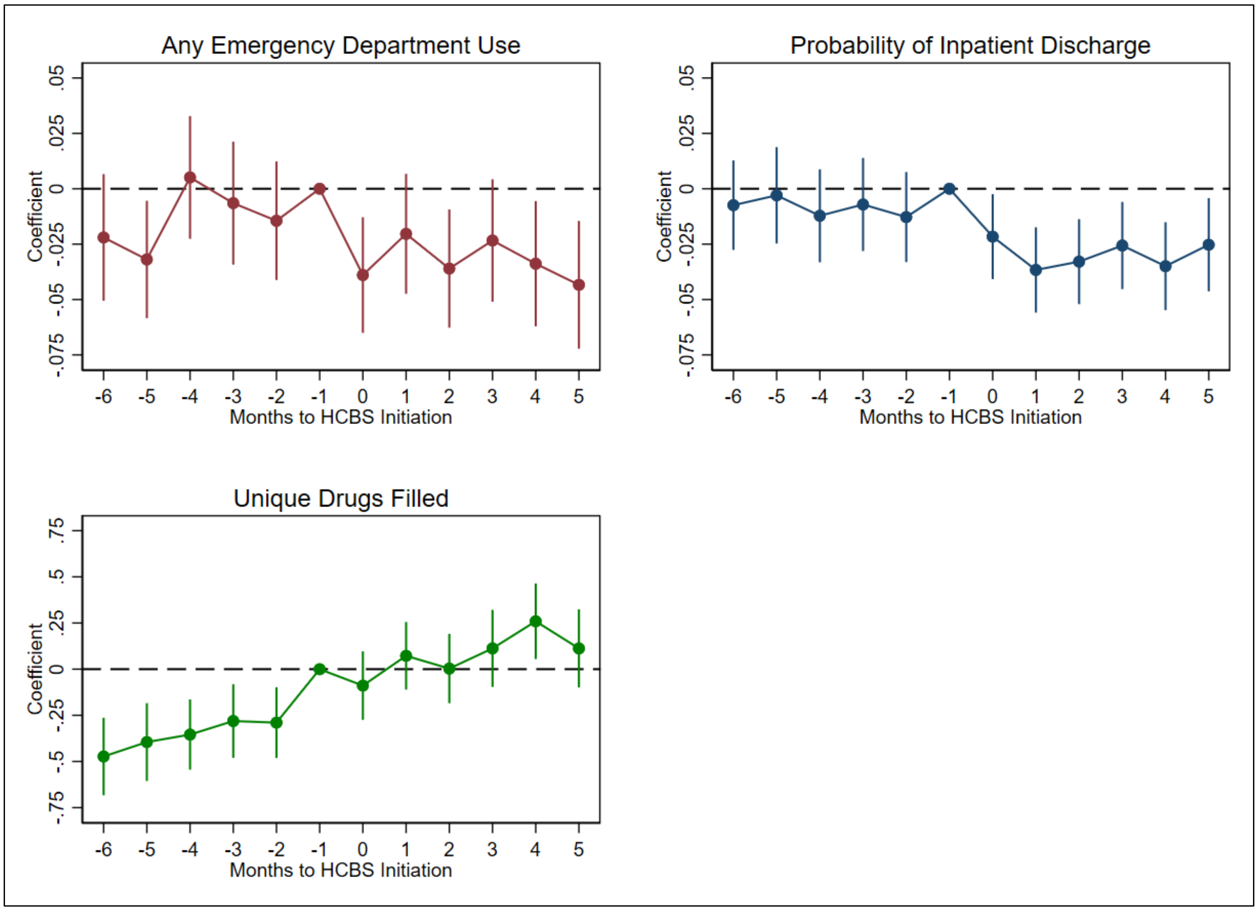

NOTE: Model sample includes individuals with any HCBS use spell. Estimates were produced using linear regression models with person-fixed effects, year fixed effects, and robust standard errors. The X axis represents time relative to HCBS initiation in person-months, where 0 is the first month in which an HCBS flag is detected. Medicare Advantage enrollees are included in the any inpatient discharge variable starting in 2010. Any emergency department use includes observation stays and outpatient admissions. Unique drugs filled include individuals with Part D for the entire observation window. HCBS = home- and community-based services.

© 2026 Keesee E et al. *JAMA Health Forum*.

eFigure 7. Dynamic Change in Outcomes Associated with HCBS Initiation Among Pre-TAF Sample

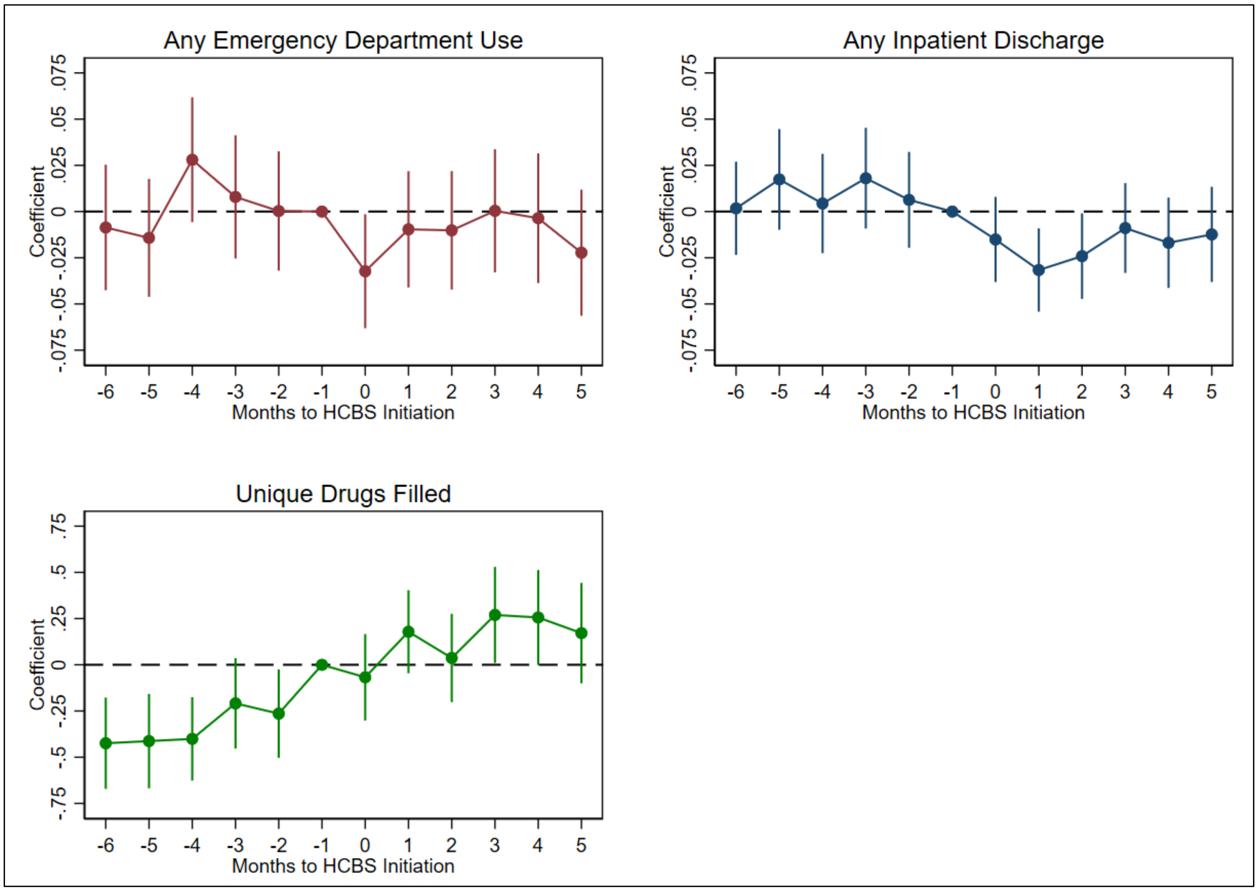

NOTE: Model sample excludes individuals with observations after their respective state's TAF transition. Estimates were produced using linear regression models with person-fixed effects, year fixed effects, and robust standard errors. The X axis represents time relative to HCBS initiation in person-months, where 0 is the first month in which an HCBS flag is detected. Medicare Advantage enrollees are included in the any inpatient discharge variable starting in 2010. Any emergency department use includes observation stays and outpatient admissions. Unique drugs filled include individuals with Part D for the entire observation window. HCBS = home- and community-based services.

eFigure 8. Dynamic Change in Outcomes Associated with HCBS Initiation when Excluding Participants with Any Nursing Home Use

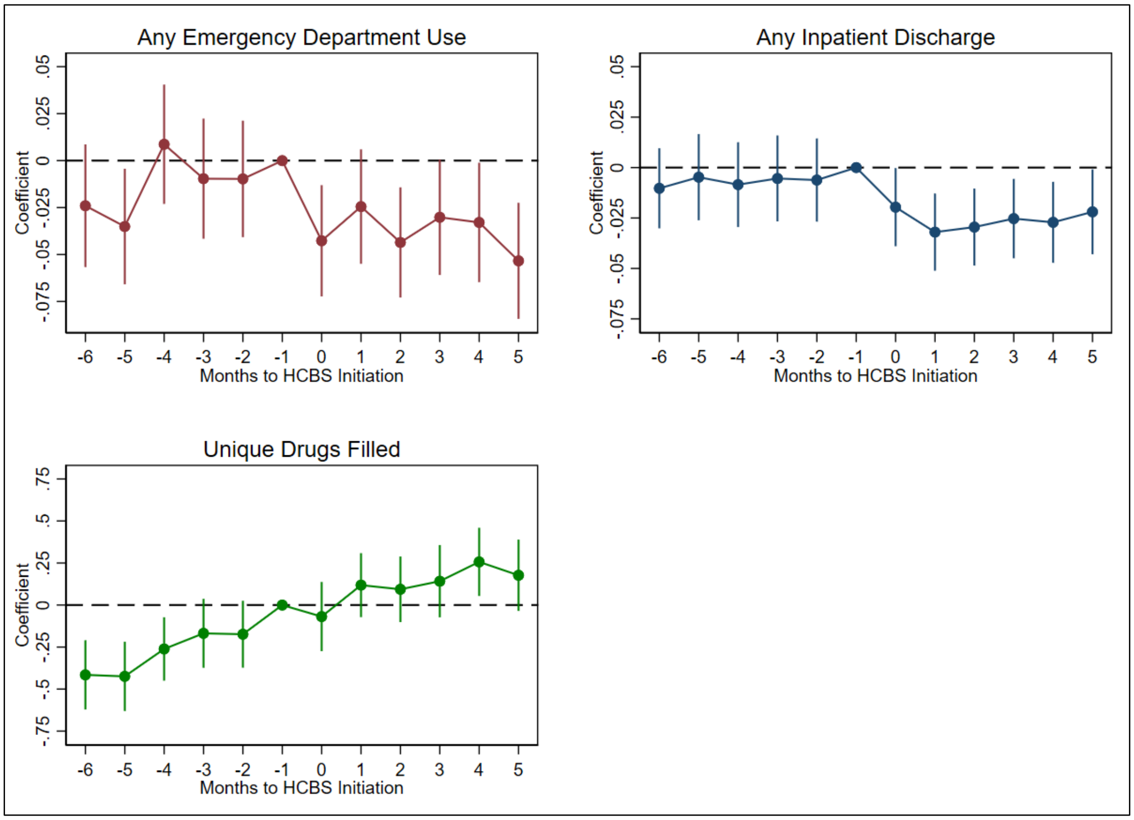

NOTE: Model sample excludes individuals without any nursing home use in the 6 months before or after HCBS initiation as indicated by an MDS record. Estimates were produced using linear regression models with person-fixed effects, year fixed effects, and robust standard errors. The X axis represents time relative to HCBS initiation in person-months, where 0 is the first month in which an HCBS flag is detected. Medicare Advantage enrollees are included in the any inpatient discharge variable starting in 2010. Any emergency department use includes observation stays and outpatient admissions. Unique drugs filled include individuals with Part D for the entire observation window. HCBS = home- and community-based services.

© 2026 Keesee E et al. *JAMA Health Forum*.

eFigure 9. Dynamic Change in Outcomes Associated with HCBS Initiation when Excluding Medicare Advantage Enrollees

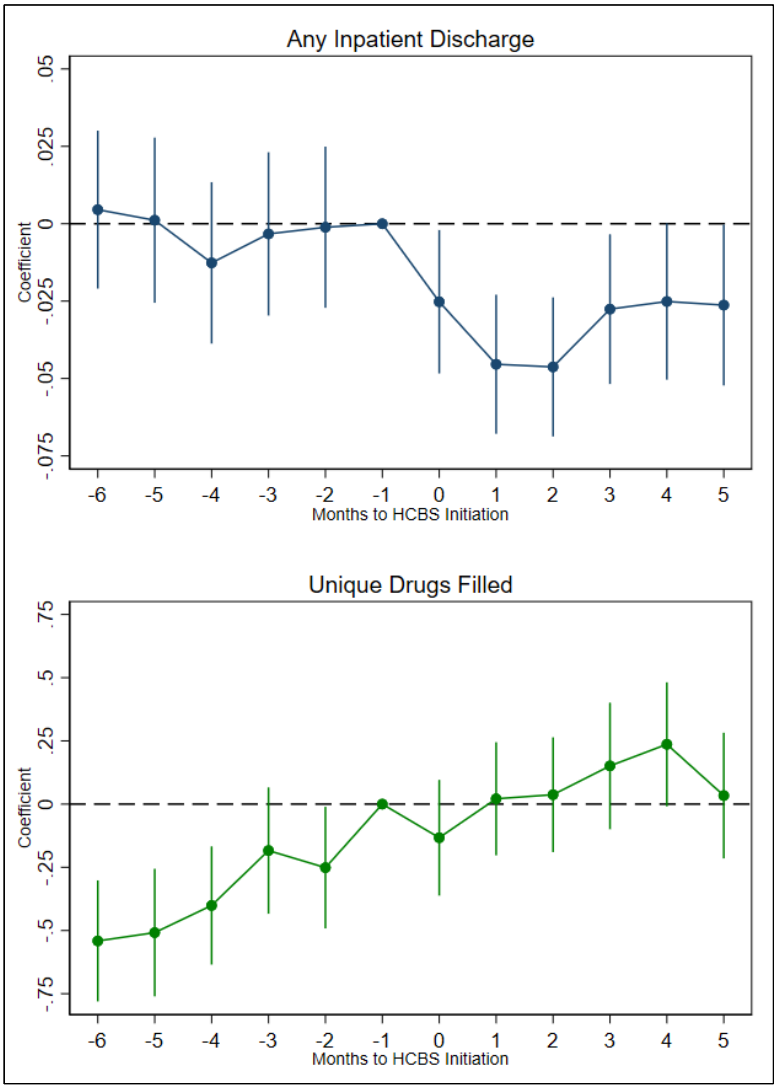

NOTE: Model sample excludes individuals enrolled in Medicare Advantage. Estimates were produced using linear regression models with person-fixed effects, year fixed effects, and robust standard errors. The X axis represents time relative to HCBS initiation in person-months, where 0 is the first month in which an HCBS flag is detected. Medicare Advantage enrollees are included in the any inpatient discharge variable starting in 2010. Any emergency department use includes observation stays and outpatient admissions. Unique drugs filled include individuals with Part D for the entire observation window. HCBS = home- and community-based services.

© 2026 Keesee E et al. *JAMA Health Forum*.

eTable 5. Pre-Post Change in Outcomes Associated with HCBS Initiation with Model Adjustments

	Any Emergency Department Use	Any Inpatient Discharge	Unique Drugs Filled
**Calendar Month-Year Fixed Effects**			
Adjusted Pre-HCBS Mean	0.118	0.083	5.756
Marginal Effect	-0.036**	-0.030***	-0.018
[95% CI]	-[0.059, -0.013]	-[0.046, -0.013]	-[0.172, 0.136]
Observations	10320	14064	14052
Unique Participants	860	1172	1171
Traditional Medicare Enrollees	X	X	X
Medicare Advantage Enrollees		X	X
Continuous Part D Coverage			X

NOTE: ***p<0.001, **p<0.01, *p<0.05. Estimates were produced using pooled pre and post outcomes and person fixed effected, year fixed effects, and robust standard errors. Medicare Advantage enrollees are included in the any inpatient discharge variable starting in 2010. Any emergency department use includes observation stays and outpatient admissions. Unique drugs filled include participants with Part D for the entire observation window. HCBS = home- and community-based services. CI = confidence interval.

© 2026 Keesee E et al. *JAMA Health Forum*.

eFigure 10. Dynamic Change in Outcomes Associated with HCBS Initiation with Month-Year Fixed Effects

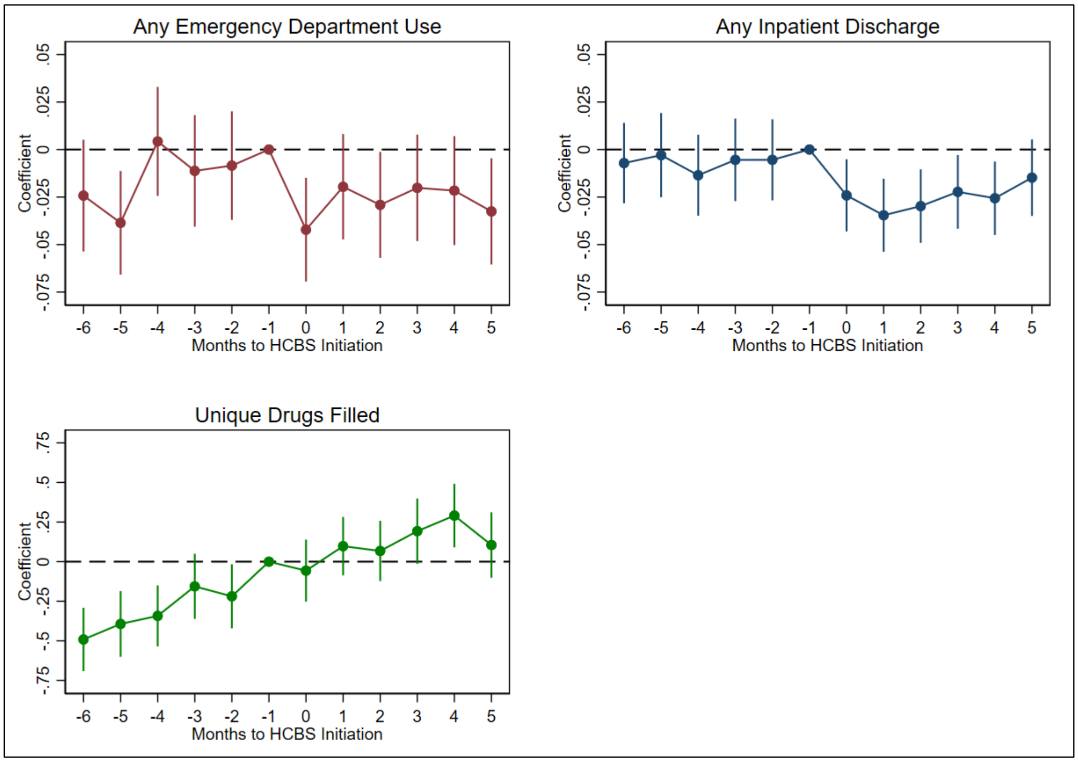

NOTE: Estimates were produced using linear regression models with person-fixed effects, month and year fixed effects, and robust standard errors. The X axis represents time relative to HCBS initiation in person-months, where 0 is the first month in which an HCBS flag is detected. Medicare Advantage enrollees are included in the any inpatient discharge variable starting in 2010. Any emergency department use includes observation stays and outpatient admissions. Unique drugs filled include individuals with Part D for the entire observation window. HCBS = home- and community-based services.

© 2026 Keesee E et al. *JAMA Health Forum*.

eFigure 11. Dynamic Change in Outcomes Resulting from State Leave One Out Models

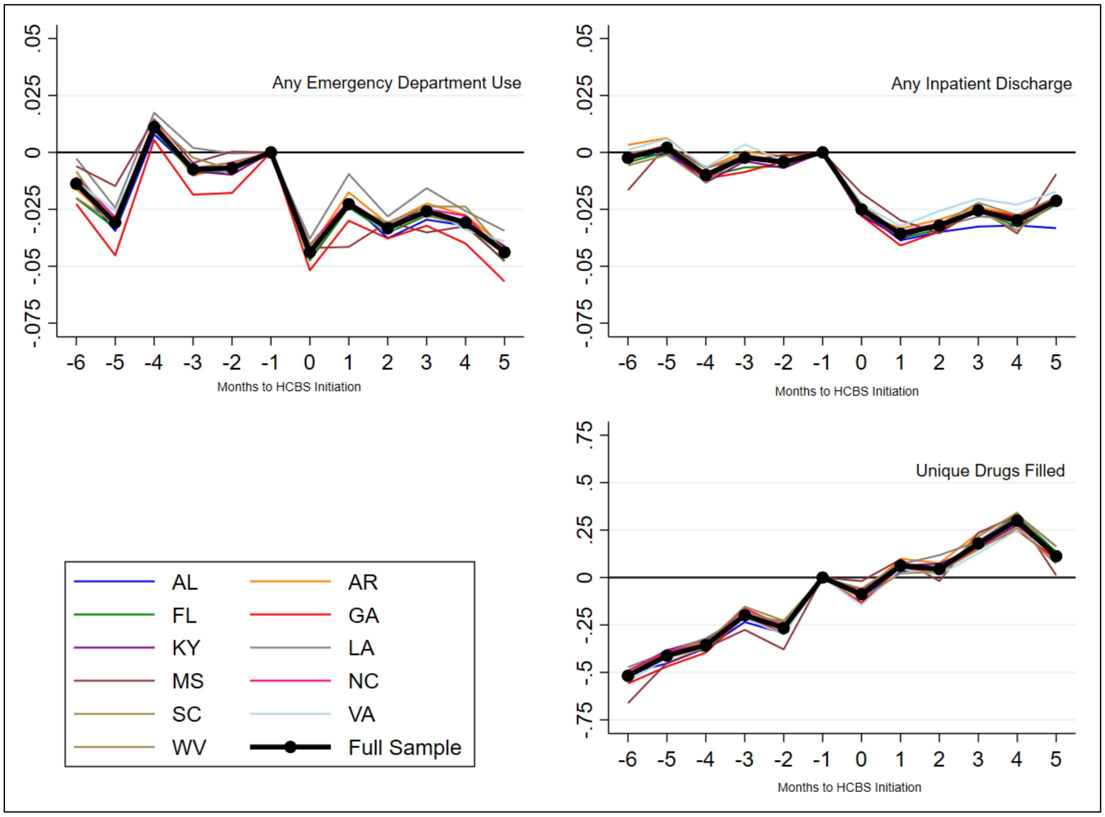

NOTE: Estimates were produced using linear regression models with person-fixed effects, year fixed effects, and robust standard errors. Each line represents the results when dropping a specified state. The horizontal axis represents time relative to HCBS initiation in person-months, where 0 is the first month in which an HCBS flag is detected. Medicare Advantage enrollees are included in the any inpatient discharge variable starting in 2010. Any emergency department use includes observation stays and outpatient admissions. Unique drugs filled include individuals with Part D for the duration of contributing person-months. HCBS = home- and community-based services.

© 2026 Keesee E et al. *JAMA Health Forum*.
