# Supplementary material for: Medicaid Home and Community-Based Services Initiation and Acute Services Use
Source: JAMA Health Forum. 2026 Mar 27;7(3):e260206. doi: 10.1001/jamahealthforum.2026.0206 (PMC13032151; doi:10.1001/jamahealthforum.2026.0206)
Supplement: Supplement 2. — Data Sharing Statement [file jamahealthforum-e260206-s002.pdf]

Data Sharing Statement

Keesee. Medicaid Home and Community-Based Services Initiation and Acute Services Use. *JAMA Health Forum*. Published March 27, 2026. doi:10.1001/jamahealthforum.2026.0206

Data
Data available: No

Additional Information
Explanation for why data not available: The use of CMS data was covered under the terms of a Data Use Agreement (DUA) and cannot be shared.
